# The association between psoriasis and nonalcoholic fatty liver disease: Mediation analysis involving inflammatory biomarkers among U.S. adults

**DOI:** 10.1371/journal.pone.0344681

**Published:** 2026-03-20

**Authors:** Xuewan Wang, Cunxiang Xie, Yutong Deng, Xuewen Ren, Yatong Li, Tangyunni Liu, Bo Hu, Huishang Feng, Yuanwen Li

**Affiliations:** 1 Beijing University of Chinese Medicine, Beijing, China; 2 Dongfang Hospital, Beijing University of Chinese Medicine, Beijing, China; 3 Shanxi Provincial Hospital of Traditional Chinese Medicine, Shanxi, China; Instituto Nacional de Ciencias Medicas y Nutricion Salvador Zubiran, MEXICO

## Abstract

**Background:**

Psoriasis has been observed to be associated with an increased risk of nonalcoholic fatty liver disease (NAFLD). However, investigating novel mediators that influence the relationship between psoriasis and NAFLD is essential, given their complex interconnection. Currently, it remains unclear whether inflammatory biomarkers play a mediating role in this association.

**Methods:**

Data from adults aged 20–60 years were obtained from the National Health and Nutrition Examination Survey (NHANES), which was conducted between 2003–2006 and 2009–2014. Logistic regression was used to analyze the association between psoriasis and NAFLD across three different models. Subgroup and interaction analyses were performed to explore potential nonlinear and independent relationships between psoriasis and NAFLD. Mediation analysis was conducted to determine whether the systemic immune-inflammation index (SII) and neutrophil-to-lymphocyte ratio (NLR), two inflammatory markers, mediate the relationship between psoriasis and NAFLD.

**Results:**

A total of 9,439 participants were included in the study, of whom 3,961 had NAFLD, with a weighted prevalence rate of 41.96%. Compared with participants without psoriasis, those with psoriasis had a greater prevalence of NAFLD, reaching 50.99%. In all three models, psoriasis was positively associated with the risk of NAFLD, and a significant dose‒response relationship was observed (p < 0.05). Mediation analysis revealed that, in the adjusted models, Ln-SII and Ln-NLR statistically mediated the association between psoriasis and NAFLD, with percentages of 8.52% and 3.58%, respectively (p < 0.05).

**Conclusion:**

Psoriasis is positively associated with the occurrence of NAFLD, and the SII and NLR play key mediating roles in the relationship between psoriasis and NAFLD.

## Introduction

Nonalcoholic fatty liver disease (NAFLD) is a chronic liver disease characterized by hepatic steatosis ≥5% without significant alcohol consumption or other known causes of liver fat accumulation [1]. It has become a public health challenge and is one of the most common chronic liver diseases worldwide. NAFLD not only directly leads to liver dysfunction and liver-related mortality but is also often accompanied by comorbidities such as obesity, type 2 diabetes, dyslipidemia, and cardiovascular diseases [2–4]. NAFLD represents a significant public health concern in the United States. Its prevalence, currently affecting an estimated 30% of Americans, has shown a consistent upward trajectory over recent decades [4,5]. Given the increasing prevalence of metabolic risk factors and an aging population, models project that the burden of advanced NAFLD-related complications will more than double by 2030 [4,6,7]. Therefore, the high prevalence and poor prognosis of NAFLD make its management particularly challenging [8], highlighting the importance of prioritizing prevention and treatment efforts, including considerations at both the clinical and population levels.

Psoriasis, a common inflammatory skin disease driven by dysregulation of both innate and adaptive immunity, affects an estimated 3% of the U.S. population. This translates to over 7.5 million individuals living with the condition [9, 10]. This disease not only involves the skin but can also lead to multisystem damage, imposing a significant burden on patients’ physical and mental health [11]. Compared with the general population, psoriasis patients are at a greater risk of developing other severe systemic diseases. A meta-analysis by Candia et al.[12] indicated that the risk of NAFLD in psoriasis patients was 2.16 times greater (95% CI: 1.19–3.93; I² = 84%) than that in nonpsoriatic individuals. The quest to identify novel factors or biomarkers for assessing the risk of severe systemic comorbidities in psoriasis patients represents a major research focus with considerable clinical promise.

Inflammatory dysregulation is a central driver in the pathogenesis of NAFLD, with hepatic lobular inflammation being a key factor in disease progression, as established in previous studies [13, 14]. Excessive fat deposition in hepatocytes triggers oxidative stress and increased inflammatory responses, a pathological process that may ultimately lead to the progression of NAFLD into cirrhosis [15]. Therefore, chronic liver inflammation is considered a critical driver of NAFLD onset and progression [13]. Notably, the characteristic systemic and local inflammatory responses in psoriasis also play a decisive role in its disease onset and progression [10]. Significant neutrophil infiltration in psoriatic lesions is a typical histological feature [16], and these cells participate in chronic inflammation through the release of cytokines, chemokines, proteases, and neutrophil elastase [17]. Monocytes, key innate immune cells, orchestrate inflammatory processes [18], while lymphocytes bridge innate and adaptive immunity [19]. Platelets contribute to maintaining homeostasis and play regulatory roles in acute and chronic inflammation, promoting the formation of the inflammatory microenvironment [20]. However, a critical gap persists in understanding the inflammatory mechanisms underlying the psoriasis-NAFLD association, making the discovery of validated biomarkers an important research objective. This underscores the growing need to identify and verify related biomarkers. On the basis of these findings, we hypothesize that psoriasis may promote the onset and progression of NAFLD by increasing systemic inflammatory marker levels. Furthermore, this in-depth exploration of inflammatory biomarkers has dual significance: on the one hand, it provides new insights into how changes in inflammation levels based on blood cells in psoriasis patients lead to the onset and progression of fatty liver; on the other hand, by evaluating the potential hepatotoxic effects of antipsoriasis medications, it can provide an important basis for the clinical development of individualized treatment plans, thereby paving the way for the precise management of psoriasis.

In summary, psoriasis and inflammation are intricately linked to NAFLD. We hypothesize (1) that psoriasis, inflammatory biomarkers, and NAFLD are correlated in pairs and (2) that inflammatory biomarkers may partially mediate the effect of psoriasis on NAFLD. Using data from the National Health and Nutrition Examination Survey (NHANES), this study had two primary aims: to evaluate the association between psoriasis and NAFLD among U.S. adults, and to investigate, via mediation analysis, the interrelationships among psoriasis, inflammatory biomarkers, and NAFLD, including the potential mediating role of these biomarkers.

## Methods

### Participants and study design

**Fig 1:**
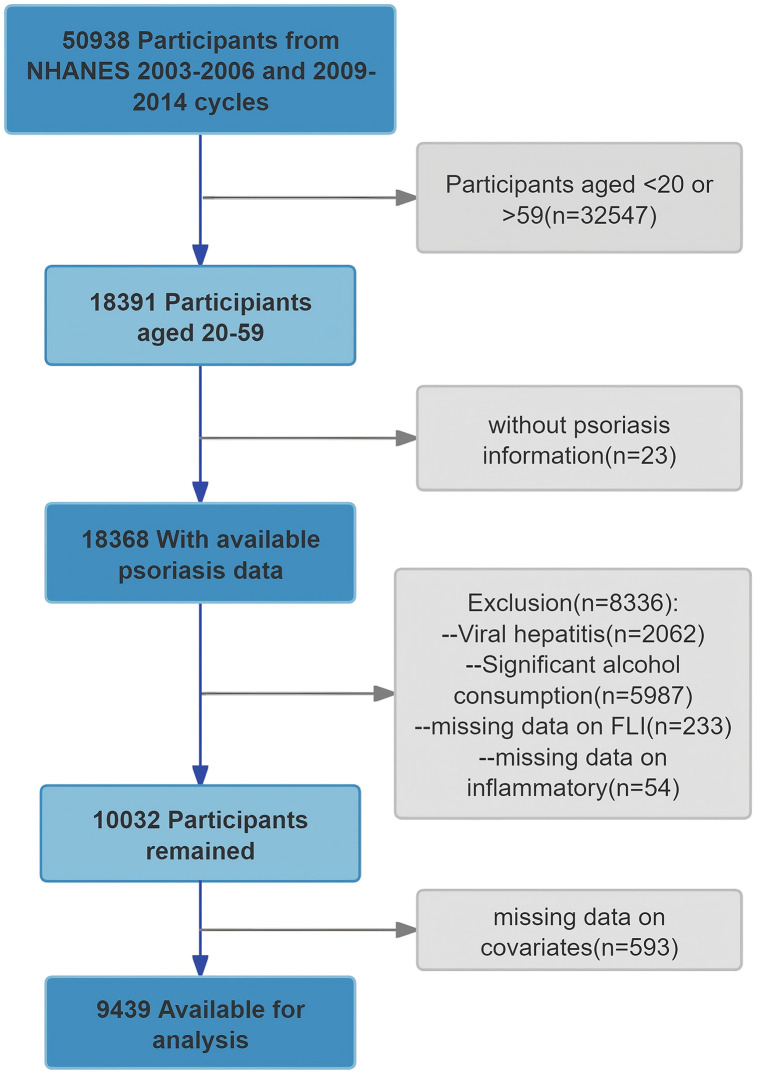
Flowchart of participants selection.

This study is a cross-sectional study based on the NHANES database [21]. During the national survey conducted in the United States, all participants provided written informed consent (https://www.cdc.gov/nchs/nhanes/index.htm). The NHANES is responsible for monitoring the health and nutritional status of the non-institutionalized civilian population in the U.S. By employing a sophisticated, stratified, multi-stage probability sampling design, NHANES ensures the collection of representative and reliable population-level data. As the present study constitutes a secondary analysis of this publicly available data, it was exempt from further ethical review and approval. A total of 50,938 participants from five NHANES cycles (2003--2004, 2005--2006, 2009--2010, 2011--2012, and 2013--2014) were included in this study. Participants were excluded if they met any of the following criteria: (1) aged 20--59 years (n = 32,547); (2) no information on psoriasis (n = 23); (3) missing data on viral hepatitis or the presence of viral hepatitis (determined by positive markers such as hepatitis B surface antigen, hepatitis C antibody, or hepatitis C RNA) (n = 2,062); missing data on alcohol consumption or excessive alcohol consumption (more than 21 drinks per week for men and more than 14 drinks per week for women) [22] (n = 5,987); missing components for FLI calculation (n = 233); missing data for inflammation calculation (n = 54); or (4) missing covariate data (n = 593). After exclusions, our final analysis included 9,439 participants (Fig 1).

### Definition of NAFLD

Owing to the lack of abdominal ultrasound data, the FLI [23], which incorporates triglycerides, BMI, γ-glutamyl transferase (GGT), and waist circumference, was used in this study to diagnose NAFLD. The calculation formula is as follows: FLI = (e^ (0.953*ln (TG) + 0.139*BMI + 0.718*ln (GGT) + 0.053*waist circumference – 15.745))/ (1 + e^ (0.953*ln (TG) + 0.139*BMI + 0.718*ln (GGT) + 0.053*waist circumference – 15.745)) * 100. An FLI threshold of ≥60 was used to indicate NAFLD [24]. For an FLI value of ≥60, the reported sensitivity, specificity, positive predictive value (PPV), and negative predictive value (NPV) were 44%, 90%, 67%, and 76%, respectively [25]. The validity of this method has been examined in previous studies [23,26].

### Assessment of psoriasis

The NHANES survey collects basic data by asking participants the following question: Has a doctor or healthcare provider ever told you that you have psoriasis? Those who answered “yes” were classified as psoriasis patients. Although the history of psoriasis was obtained from participants’ self-reports, previous studies have validated the reliability of self-reported psoriasis history [27].

### Measurement of inflammatory biomarkers

In this study, the inflammatory biomarkers of the subjects involved in the examination were the neutrophil-to-lymphocyte ratio (NLR) and the systemic immune-inflammation index (SII). The SII is a novel biomarker of the systemic local immune response and systemic inflammation and was analyzed via the Beckman Coulter HMX hematology analyzer on peripheral blood samples from NHANES participants at the Mobile Examination Center (MEC); the SII is expressed as ×10³ cells/μL, with detailed standardization protocols provided in the NHANES Laboratory Procedures Manual. The SII was calculated as the platelet count * neutrophil count/lymphocyte count [28].

### Covariate assessment

A multivariate adjustment model was constructed to explore the impact of confounding variables on the relationship between psoriasis and nonalcoholic fatty liver disease, and the covariates in this study spanned comprehensive sociodemographic characteristics, lifestyle habits, and chronic disease history across multiple dimensions. These include age (years), sex (male or female), race, education level, marital status, the family poverty income ratio (PIR), smoking status, and a history of chronic diseases such as diabetes and coronary heart disease.

Race was categorized as Mexican American, non-Hispanic White, non-Hispanic Black, other Hispanic, or other races. Education level was classified as less than high school, high school or equivalent, or college or above. Marital status was divided into married, unmarried, living with a partner, and other (including widowed, divorced, or separated). To classify family income, participants were categorized as low income (PIR ≤ 1.3), middle income (1.3 < PIR ≤ 3.5), or high income (PIR > 3.5) according to PIR standards defined in U.S. government reports. Regarding lifestyle habits, smoking status was classified as never smoker (smoking fewer than 100 cigarettes in their lifetime), current smoker (smoking at least 100 cigarettes in their lifetime and continuing to smoke), or former smoker (smoking at least 100 cigarettes in their lifetime but no longer smoking). With respect to medical history, diabetes was defined as a self-reported history of diabetes, fasting plasma glucose (FPG) level ≥ 7.0 mmol/L, 2-hour postload glucose level ≥ 11.0 mmol/L, HbA1c ≥ 6.5%, or the use of antidiabetic medications or insulin. The history of coronary heart disease was determined on the basis of whether a doctor had informed the participant that they had these conditions, recorded as binary variables (yes or no).

### Statistical analysis

The data were weighted according to the NHANES standard sample weighting guidelines to ensure representativeness. Continuous variables are presented as the means ± standard deviations (SDs), and P values were calculated via weighted linear regression models. Categorical variables are described as N (%), and P values were calculated via weighted chi-square tests. Multivariate logistic regression analyses were conducted to examine the association between psoriasis and NAFLD, and 95% confidence intervals (CIs) and odds ratios (ORs) were calculated. Three models were constructed to adjust for confounding factors. Model 1 was a crude model without adjustment for confounders; Model 2 was a partially adjusted model that accounted for age, sex, race, education level, marital status, and PIR. Model 3 was a fully adjusted model that further accounted for smoking status, diabetes, and coronary heart disease. Additionally, interaction and subgroup analyses were performed via logistic regression models based on age, sex, race, education level, marital status, PIR, smoking status, diabetes, and coronary heart disease.

To test the robustness of these findings, we conducted a sensitivity analysis based on the Hepatic Steatosis Index (HSI), which has been utilized and validated in numerous epidemiological studies [29,30]. The HSI was calculated as follows: HSI = 8 × (ALT [IU/L]/ AST [IU/L]) + body mass index (BMI, kg/m²) + 2 (if female) + 2 (if having type 2 diabetes). According to previous publications, participants with an HSI > 36 were defined as having NAFLD [31].

Next, we explored the associations between inflammatory biomarkers and psoriasis and NAFLD. Multivariable linear regression was used to analyze the associations between psoriasis and the SII and NLR, whereas multivariable logistic regression was employed to investigate the relationships between the SII and the NLR and NAFLD. Notably, during the statistical analysis, we observed a significant right-skewed distribution of the SII and NLR data and thus applied natural logarithm transformation (Ln, natural logarithm) to make the data more suitable for our statistical analysis.

**Fig 2:**
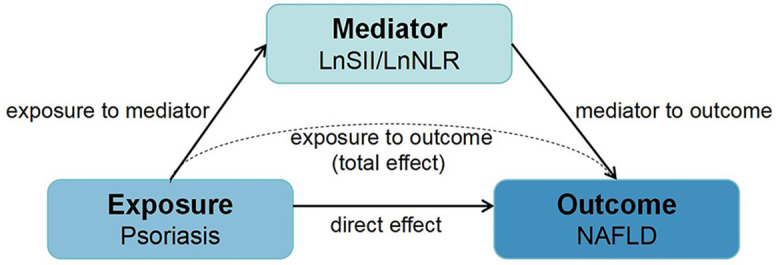
Path diagram of the mediation analysis models.

To investigate whether inflammatory biomarkers mediate the association between psoriasis and NAFLD, we performed mediation analysis using the “mediation” package in R. As shown in Fig 2, the mediation model includes the exposure to the mediator, the mediator to the outcome, and the exposure to the outcome (total effect) [32]. In this study, the direct effect represents the association between psoriasis and NAFLD; the indirect effect, i.e., the association between psoriasis and NAFLD, is mediated by inflammatory biomarkers. The total effect is the sum of the direct effect and the indirect effect, and the proportion of the mediation effect is calculated as the indirect effect/total effect × 100%. All the statistical analyses were performed via R (version 4.4.2, R Foundation for Statistical Computing, Vienna, Austria), and a P value < 0.05 (two-tailed) was considered statistically significant.

## Results

### Characteristics of the study population

We identified 9,439 participants. A total of 253 participants (2.68%) had psoriasis, whereas 9,186 participants (97.32%) did not have psoriasis. Among them, 41.96% of the participants had FLI ≥ 60, and 58.04% had FLI < 60. Table 1 presents the weighted characteristics of the study population. Male participants accounted for 51.26%, and those aged 20--39 years accounted for 55.42%. Notably, the prevalence of NAFLD among psoriasis participants was 50.99%, which was higher than the 41.72% reported in non-psoriasis participants. Furthermore, participants with NAFLD were more likely to be Mexican American (52.38% vs. 39.95%), have an education level of less than high school (53.4% vs. 41.34%), be widowed, divorced, or separated (48.52% vs. 40.93%), or have a middle-to-low income level (44.18% vs. 38.32%). Statistically significant differences (P < 0.05) were observed between the two groups in terms of age, sex, race, education level, marital status, income level, smoking status, diabetes status, coronary heart disease status, the SII, and the NLR.

**Table 1:** Characteristics of the study population (weighted).

Variable	Total (n = 9439)	Non-NAFLD (n = 5478)	NAFLD (n = 3961)	P-value
**Gender**				<0.001
**Male**	4838 (51.26)	2508 (45.78)	2330 (58.82)	
**Female**	4601 (48.74)	2970 (54.22)	1631 (41.18)	
**Age**				<0.001
**20-39**	5231 (55.42)	3382 (61.74)	1849 (46.68)	
**40-59**	4208 (44.58)	2096 (38.26)	2112 (53.32)	
**Race/ethnicity**				<0.001
**Mexican American**	1531 (16.22)	729 (13.31)	802 (20.25)	
**Other Hispanic**	695 (7.36)	409 (7.47)	286 (7.22)	
**Non-Hispanic White**	4546 (48.16)	2729 (49.82)	1817 (45.87)	
**Non-Hispanic Black**	1817 (19.25)	1011 (18.46)	806 (20.35)	
**Other Race – Including Multi-Racial**	850 (9.01)	600 (10.95)	250 (6.31)	
**Education Levels**				<0.001
**Less than high school**	485 (5.14)	226 (4.13)	259 (6.54)	
**high school or equivalent**	3160 (33.48)	1663 (30.36)	1497 (37.79)	
U**niversity and above**	5794 (61.38)	3589 (65.52)	2205 (55.67)	
**Marital Status**				<0.001
**Married**	4771 (50.55)	2622 (47.86)	2149 (54.25)	
**Never married**	2371 (25.12)	1572 (28.7)	799 (20.17)	
**Living with partner**	1009 (10.69)	621 (11.34)	388 (9.8)	
**Other**	1288 (13.65)	663 (12.1)	625 (15.78)	
**Poverty income ratio**				0.004
**Low income**	2629 (27.85)	1475 (26.93)	1154 (29.13)	
**middle income**	3235 (34.27)	1798 (32.82)	1437 (36.28)	
**high income**	3575 (37.87)	2205 (40.25)	1370 (34.59)	
**Psoriasis**				0.01
**No**	9186 (97.32)	5354 (97.74)	3832 (96.74)	
**Yes**	253 (2.68)	124 (2.26)	129 (3.26)	
**Smoking**				<0.001
**Never smoked**	5221 (55.31)	3188 (58.2)	2033 (51.33)	
**Current smoker**	1915 (20.29)	1102 (20.12)	813 (20.53)	
**Former smoker**	2303 (24.40)	1188 (21.69)	1115 (28.15)	
**Diabetes**				<0.001
**No**	8704 (92.21)	5317 (97.06)	3387 (85.51)	
**Yes**	735 (7.79)	161 (2.94)	574 (14.49)	
**Cornoary heart disease**				<0.001
**No**	9355 (99.11)	5447 (99.44)	3908 (98.66)	
**Yes**	84 (0.89)	31 (0.56)	53 (1.34)	
**SII**	552.31 ± 318.83	534.96 ± 315.23	577.31 ± 322.28	<0.001
**NLR**	2.15 ± 1.04	2.12 ± 1.06	2.19 ± 1	0.007

Mean±SD for continuous variables, N (%) for categorical variables.

SII, systemic immune-inflammation index; NLR, neutrophil-to-lymphocyte ratio; Ln, natural logarithm.

### Association between psoriasis and NAFLD

Logistic regression was used to assess the relationship between psoriasis risk score and NAFLD risk score, and the results are shown in Table 2. Evidently, a significant positive correlation was observed across all the models. Baseline Model 1 revealed that the risk of NAFLD in psoriasis patients increased significantly, by 49% (95% CI: 1.093, 2.033; p = 0.012). After adjusting for demographic variables (age, sex, race, education level, marital status, PIR), the risk ratio in Model 2 decreased slightly to 49.5% (95% CI: 1.070, 2.089; p = 0.019). Finally, after further adjustment for clinical variables (smoking status, diabetes, and history of CHD), Model 3 still demonstrated a 50.8% increased risk (95% CI: 1.080, 2.104; p = 0.017). These results consistently indicate a stable and significant association between psoriasis and NAFLD risk.

**Table 2:** Association between psoriasis and NAFLD (weighted).

	Model1		Model2		Model3	
	OR (95%CI)	P value	OR (95%CI)	P value	OR (95%CI)	P value
**Without psoriasis**	Ref.					
**With psoriasis**	1.490 (1.093, 2.033)	**0.012**	1.495 (1.070, 2.089)	**0.019**	1.508 (1.080, 2.104)	**0.017**

Model 1: Not adjusted.

Model 2: Adjusted for age, sex, race, education level, marital status, and PIR.

Model 3: Additionally adjusted for age, sex, race, education level, marital status, PIR, smoking, diabetes, and coronary heart disease.

NAFLD, non-alcoholic fatty liver disease; Ref., reference.

Furthermore, as shown in S1 Table, the association between psoriasis and NAFLD persisted in the sensitivity analysis when NAFLD was defined by HSI (OR, 1.483; 95% CI, 1.133–1.942). In the fully adjusted model, the association between psoriasis and NAFLD remained largely unchanged (OR, 1.559; 95% CI, 1.191–2.041).

### Subgroup analysis

**Fig 3:**
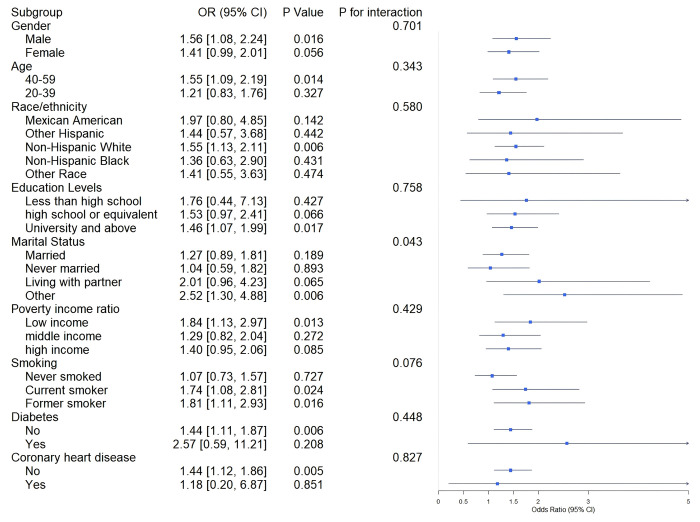
Association between psoriasis and NAFLD by different group stratification.

To explore the prevalence of the association between psoriasis and NAFLD across different populations, we conducted a subgroup analysis. As shown in Fig 3, with nine stratification factors included in the analysis. The results revealed a significant positive correlation between psoriasis and NAFLD occurrence among males, individuals aged 40–59 years, non-Hispanic whites, those who were widowed/separated/divorced, low-income groups, current smokers and former smokers, and individuals without diabetes or coronary heart disease. However, the association between psoriasis and NAFLD occurrence was not significant in other populations. Interaction tests revealed no significant dependency of the positive correlation between psoriasis and NAFLD on sex, age, race, education, smoking status, diabetes status, or coronary heart disease (P for interaction >0.05). Only in the marital status stratification did the association between psoriasis and NAFLD significantly differ (P for interaction = 0.043).

### Inflammatory biomarkers and their associations with psoriasis and NAFLD

Table 3 shows the associations between psoriasis and inflammatory biomarkers. The results of weighted multivariate linear regression indicate a significant positive correlation between psoriasis and ln-SII and ln-NLR across all the models. In the fully adjusted model (Model 3), each unit increase in psoriasis risk corresponded to an 8.7% increase in ln-SII risk (β = 0.087, 95% CI: 0.017, 0.158, P = 0.016) and a 4.4% increase in ln-NLR risk (β = 0.044, 95% CI: 0.006, 0.083, P = 0.024).

**Table 3:** Association between psoriasis and inflammatory biomarkers (weighted).

Variable	Model1		Model2		Model3	
	β (95%CI)	P value	β (95%CI)	P value	β (95%CI)	P value
**LnSII**	0.092 (0.022,0.162)	**0.011**	0.088 (0.019,0.158)	**0.014**	0.087 (0.017,0.158)	**0.016**
**LnNLR**	0.048 (0.009,0.086)	**0.017**	0.044 (0.006,0.082)	**0.023**	0.044 (0.006,0.083)	**0.024**

Model 1: Not adjusted.

Model 2: Adjusted for age, sex, race, education level, marital status, and PIR.

Model 3: Additionally adjusted for age, sex, race, education level, marital status, PIR, smoking, diabetes, and coronary heart disease.

SII, systemic immune-inflammation index; NLR, neutrophil-to-lymphocyte ratio; Ln, natural logarithm.

Table 4 summarizes the relationships between inflammatory markers and NAFLD. Weighted multivariable logistic regression analysis revealed a positive correlation between inflammatory markers and the risk of NAFLD in all the models. When inflammatory markers were considered continuous variables, in the fully adjusted model (Model 3), each unit increase in ln-SII was associated with a 54% increased risk of NAFLD (OR: 1.54, 95% CI: 1.37, 1.72, P < 0.001), and each unit increase in ln-NLR was associated with a 34% increased risk of NAFLD (OR: 1.34, 95% CI: 1.10, 1.62, P = 0.003).

**Table 4:** Association between inflammatory biomarkers and NAFLD (weighted).

Variable	Model1		Model2		Model3	
	OR (95%CI)	P value	OR (95%CI)	P value	OR (95%CI)	P value
**LnSII**	1.42 (1.27, 1.58)	**< 0.001**	1.55 (1.37, 1.74)	**< 0.001**	1.54 (1.37, 1.72)	**< 0.001**
**LnNLR**	1.41 (1.18, 1.69)	**< 0.001**	1.38 (1.14, 1.67)	**0.001**	1.34 (1.10, 1.62)	**0.003**

Model 1: Not adjusted.

Model 2: Adjusted for age, sex, race, education level, marital status, and PIR.

Model 3: Additionally adjusted for age, sex, race, education level, marital status, PIR, smoking, diabetes, and coronary heart disease.

SII, systemic immune-inflammation index; NLR, neutrophil-to-lymphocyte ratio; Ln, natural logarithm.

### Mediation effects of inflammatory biomarkers

We analyzed the relationship between psoriasis and the risk of NAFLD using inflammatory biomarkers as mediating variables. As shown in Table 5, the results indicated that both SII and NLR exhibited significant mediating effects in the association between psoriasis and NAFLD. In the analyses using LnSII and LnNLR as mediators, we observed that they both partially mediated the relationship between psoriasis and NAFLD. In Model 1, the Ln-SII explained 7.26% of the association (P = 0.005), and the Ln-NLR explained 3.65% (P = 0.008) of the association. In Model 2, Ln-SII accounted for 9.09% of the associations (P = 0.008), and Ln-NLR accounted for 4.1% (P = 0.01). In Model 3, the mediation proportions were 8.52% (P = 0.011) and 3.58% (P = 0.014), respectively.

**Table 5:** Mediating effects of inflammatory biomarkers on the association between psoriasis and NAFLD.

Variable		ACME	Total effect	Mediation_Proportion	P_Value
		β (95%)	β (95%)		
**LnSII**	Model1	0.007 (0.002, 0.012)	0.093 (0.031, 0.156)	7.26%	**0.005**
	Model2	0.008 (0.002, 0.014)	0.085 (0.025, 0.144)	9.09%	**0.008**
	Model3	0.007 (0.002, 0.013)	0.082 (0.025, 0.141)	8.52%	**0.011**
**LnNLR**	Model1	0.003 (0.001, 0.007)	0.093 (0.031, 0.156)	3.65%	**0.008**
	Model2	0.004 (0.001, 0.007)	0.085 (0.026, 0.144)	4.10%	**0.01**
	Model3	0.003 (0.001, 0.006)	0.083 (0.026, 0.141)	3.58%	**0.014**

Model 1: Not adjusted.

Model 2: Adjusted for age, sex, race, education level, marital status, and PIR.

Model 3: Additionally adjusted for age, sex, race, education level, marital status, PIR, smoking, diabetes, and coronary heart disease.

SII, systemic immune-inflammation index; NLR, neutrophil-to-lymphocyte ratio; Ln, natural logarithm; ACME, Average Causal Mediation Effects.

## Discussion

To our knowledge, the current cross-sectional study is the first to investigate the mediating role of psoriasis on the risk of NAFLD in a large nationally representative sample. We found that psoriasis is a risk factor for NAFLD, and inflammatory biomarkers serve as mediators between psoriasis and NAFLD.

In this study, the weighted prevalence of NAFLD was 41.96%, which was slightly higher than the 32.4% reported in an epidemiological study in 2022 [33]. This difference may be related to the exclusion of individuals with missing NAFLD-related data (e.g., viral hepatitis, alcohol consumption history) during our analysis. Additionally, the prevalence of NAFLD among psoriasis patients was higher, reaching 50.99%, which was also slightly higher than that reported in a cohort study based on ultrasound diagnosis conducted in Houston, Texas (46.6%) [34]. This may reflect the limited sensitivity of ultrasound in detecting mild steatosis. Previous meta-analyses have shown a positive correlation between psoriasis and NAFLD [12], which is consistent with our findings. In logistic regression analysis, after adjusting for variables related to sociodemographic characteristics, lifestyle habits, and chronic disease history, psoriasis remained significantly associated with NAFLD. In subgroup analyses, we found that a positive correlation between psoriasis and NAFLD existed among males, individuals aged 40–59 years, non-Hispanic White individuals, those who were widowed/separated/divorced, low-income individuals, current smokers and former smokers, individuals without diabetes, and those without CHD. These observations imply that the psoriasis-NAFLD association may involve additional confounders, necessitating interventional or experimental studies to elucidate the underlying causal mechanisms. Notably, a significant association between psoriasis and NAFLD risk score was observed among widowed/separated/divorced individuals, and the OR for the association between psoriasis and NAFLD was greater in psoriasis patients with diabetes.

A substantial body of evidence indicates that changes in peripheral blood neutrophils, monocytes, lymphocytes, and platelets are associated with psoriasis [35–37]. In recent years, Hu et al. developed a novel inflammatory marker, the SII [38], which provides a superior tool for studying systemic immune-inflammatory states. These inflammation markers, which are based on routine blood tests (such as the SII and NLR), not only offer the advantages of low cost and convenient detection but also provide reliable and reproducible information for systemic inflammation assessment. As an emerging inflammatory marker, the SII integrates neutrophil, platelet, and lymphocyte counts to effectively reflect the balance between inflammation and the immune response, thereby providing an objective evaluation of individual inflammation levels and immune activity [28,39]. This study found that patients with psoriasis had significantly elevated levels of SII and NLR, which further supports the association between psoriasis and a heightened systemic inflammatory state. Furthermore, SII and NLR levels were also significantly higher in subjects with NAFLD compared to those without NAFLD, and the observed trends suggested a dose-response relationship. These associations remained statistically significant after multivariate adjustment, consistent with previous findings [40]. These discoveries suggest that inflammatory biomarkers, such as SII and NLR, may serve as potential indicators for assessing the risk of NAFLD.

Therefore, to explore the potential inflammation-mediated mechanisms between psoriasis and NAFLD, we conducted a mediation analysis. Notably, A significant positive association was observed between psoriasis severity and NAFLD risk. Although this association was partially mediated by inflammatory indicators, with SII and NLR accounting for 8.52% and 3.58% of the effect, respectively, it is crucial to emphasize that the modest proportion of mediation suggests that the inflammatory pathway may play a role, but the majority of the association remains unexplained by these biomarkers. These findings not only deepen our understanding of the pathogenesis of psoriasis-related NAFLD but also, more importantly, reveal a potential pathophysiological pathway linking the two diseases through systemic inflammation. Biomarkers derived from routine blood tests, such as SII and NLR, offer a practical tool and a feasible framework for the clinical assessment and management of NAFLD risk in psoriatic patients.

Our study has several notable strengths. First, it utilizes the nationally representative NHANES population, and the large sample size supports the reliability of our research. Second, we adjusted for confounding factors, including sociodemographic characteristics, lifestyle habits, and chronic disease history, to produce more reliable results. Furthermore, this study is the first to attempt to establish the relationship between psoriasis and NAFLD through inflammation by introducing blood cell-based inflammatory biomarkers as mediators. The use of different indicators allows for a multifaceted evaluation of inflammation from various perspectives, making our analysis more comprehensive. Importantly, the use of common methods to measure the SII and NLR makes them accessible and low-cost biomarkers with potential clinical utility. Considering that inflammation status appears to be modifiable through psoriasis control, understanding the impact of blood cell-based inflammatory biomarkers on NAFLD has further health implications, supporting early identification and prevention of NAFLD.

This study has several limitations. First, its cross-sectional design precludes causal inference between psoriasis and NAFLD. Second, psoriasis was defined based on self-report questionnaires, which may introduce recall bias. Although the large sample size and complex multistage sampling design partially compensate for this limitation, previous studies have shown that self-reported psoriasis has high specificity [27,41], and self-reported data may underestimate the prevalence of psoriasis [42], which could lead to an attenuation of the OR for the association between psoriasis and NAFLD in our study. Additionally, the FLI was used in our study as a noninvasive risk score to predict NAFLD, rather than imaging methods commonly used in clinical practice. However, this index has been validated across diverse populations as a simple, cost-effective tool suitable for large-scale epidemiological and clinical use [43,44]. It is not without limitations. Furthermore, the sample size of psoriasis patients (n = 253), although sufficient for the primary analysis, posed limitations for the subgroup analyses. The limited power, combined with the lack of adjustment for multiple testing, increases the risk of misinterpretation (including both Type I and Type II errors), which likely explains the non-significance of most interaction tests. Therefore, the subgroup findings should be interpreted as exploratory and hypothesis-generating, requiring validation in larger studies. Finally, despite adjusting for a range of known confounders, the possibility of residual confounding cannot be ruled out. This is due to the lack of data on unmeasured or incompletely measured factors in NHANES, such as detailed genetic information, lifestyle factors (e.g., diet, physical activity), and certain obesity-related metabolic details. To maximize the sample size and avoid selection bias introduced by missing data on these variables, we deliberately excluded them from the primary analysis model. While this trade-off was necessary, it may have compromised the completeness of confounding control. Therefore, although evidence regarding the relationship between psoriasis and NAFLD is emerging, the underlying mechanisms remain far from conclusive. On the basis of our work, future prospective studies incorporating larger sample sizes and more comprehensive data are warranted to further investigate the inflammatory effects of psoriasis and better delineate its association with NAFLD.

## Conclusion

In summary, our study confirms that the blood cell-based inflammatory biomarkers NLR and the SII mediate the association between psoriasis and NAFLD. These markers are easy to detect, cost-effective, and hold significant clinical value. In the clinical management of patients with psoriasis, early diagnosis, regular monitoring, individualized treatment plans to reduce drug hepatotoxicity, and the integration of inflammatory marker assessments should prioritize inflammation management strategies. This comprehensive approach can help identify high-risk individuals for NAFLD early, thereby reducing the risk of adverse outcomes associated with NAFLD.

## Supporting information

S1 Table Sensitivity Analyses. Model 1: Not adjusted. Model 2: Adjusted for age, sex, race, education level, marital status, and PIR. Model 3: Additionally adjusted for age, sex, race, education level, marital status, PIR, smoking, diabetes, and coronary heart disease. HSI, Hepatic Steatosis Index; Ref., reference.
(DOCX)

S1 File Minimal data set. (DOCX)
