# Supplementary material for: The association between psoriasis and nonalcoholic fatty liver disease: Mediation analysis involving inflammatory biomarkers among U.S. adults
Source: PLoS One. 2026 Mar 20;21(3):e0344681. doi: 10.1371/journal.pone.0344681 (PMC13004334; doi:10.1371/journal.pone.0344681)
Supplement: S1 Table — Model 1: Not adjusted. Model 2: Adjusted for age, sex, race, education level, marital status, and PIR. Model 3: Additionally adjusted for age, sex, race, education level, marital status, PIR, smoking, diabetes, and coronary heart disease. HSI, Hepatic Steatosis Index; Ref., reference. (DOCX) [file pone.0344681.s001.docx]

**Table S1. Sensitivity Analyses**

| **NAFLD defined by HSI score** | **Model1** | | **Model2** | | **Model3** | |
| --- | --- | --- | --- | --- | --- | --- |
|  | OR(95%CI) | P value | OR(95%CI) | P value | OR(95%CI) | P value |
| **Without psoriasis** | Ref. | | | | | |
| **With psoriasis** | 1.483(1.133, 1.942) | **0.005** | 1.553(1.188, 2.030) | **0.002** | 1.559(1.191, 2.041) | **0.002** |

Model 1: Not adjusted

Model 2: Adjusted for age, sex, race, education level, marital status, and PIR

Model 3: Additionally adjusted for age, sex, race, education level, marital status, PIR, smoking, diabetes, and coronary heart disease

HSI, Hepatic Steatosis Index; Ref., reference
