# Supplementary material for: The association between psoriasis and nonalcoholic fatty liver disease: Mediation analysis involving inflammatory biomarkers among U.S. adults
Source: PLoS One. 2026 Mar 20;21(3):e0344681. doi: 10.1371/journal.pone.0344681 (PMC13004334; doi:10.1371/journal.pone.0344681)
Supplement: S1 File — (DOCX) [file pone.0344681.s002.docx]

DOI：10.6084/m9.figshare.30229150

https://figshare.com/articles/dataset/Minimal_data_set_/30229150?file=58339180
